# Assessing the capacity and findings of routine programmatic data in Kenya to guide decision-making around contraceptives and antiretroviral therapy

**DOI:** 10.1186/s12916-021-02066-6

**Published:** 2021-08-13

**Authors:** Beth A. Tippett Barr

**Affiliations:** U.S. Centers for Disease Control and Prevention, Kisumu, Kenya

**Keywords:** HIV, Prevention of mother-to-child transmission (PMTCT), Contraceptives, Efficacy, Integrated services, Electronic medical records (EMR), Program data, Surveillance, Safety signals, Drug interactions

## Background

The World Health Organization’s comprehensive strategic approach to prevention of mother-to-child transmission of HIV (PMTCT) covers the “4-prongs” of PMTCT programming: primary prevention of HIV; prevention of unintended pregnancies; prevention of vertical transmission from mother to infant; and provision of treatment, care, and support to mothers and infants [1, 2].

In resource-constrained settings, ensuring universal access to contraceptive options can be logistically challenging, and this is further complicated by the potentially reduced effectiveness of some long-term contraceptives in the presence of specific antiretrovirals. Clinician and patient contraceptive and antiretroviral therapy (ART) decisions are made within the larger context of standardized national guidelines and standardized essential medicines procurement at the national level, which, while simplifying procurement and improving cost-efficiency for the national health system [3], may not allow for a wide array of options available to individual patients.

This provision of ART and contraceptives becomes particularly problematic in situations where negative drug interactions are reported *after* the national introduction of new guidelines and commodities. Patel and colleagues [4] refer to the recent global “scare” about a potential association between dolutegravir use in pregnancy and neural tube defects [5] and how the subsequent rapid transition of some national programs to an efavirenz (EFV)-based ART regimen had the undesired knock-on effect of potentially reducing effectiveness of long-term contraceptive use in women who were on EFV, which they had reported in an earlier paper [6].

In this paper, Patel and colleagues comment on Kenya’s “robust national electronic medical record (EMR) system” [4] and follow-up their previous paper [6] by conducting a study with the dual intent of (1) conducting “a three-phase validation study” on the ability of strong national EMRs to provide accurate and reliable data to guide decision-making and (2) to better estimate the associations between long-term contraceptive methods, EFV-containing ART regimens, and incident pregnancy [4].

The three-phase validation included EMR review of 4 years of records from over 85,000 women living with HIV (WLHIV) (> 170,000 person-years of observation), 5000 random chart abstractions, and 1000 phone interviews with women who did and did not become pregnant using EFV and long-term contraceptive methods. Using these methods, Patel and colleagues planned to pool program data to determine if there is reduced contraceptive effectiveness in a real-world setting, while simultaneously confirming that the national program data used for analysis is reliable enough to guide future decision-making around contraceptive choices and ART regimens.

## Findings

### Data validation

On the data-validation objective of the study, Patel demonstrated that all three sampling methods provided overlapping confidence intervals, or non-significant variation, on the associations between long-term contraceptive methods including implants and depomedroxyprogesterone acetate (DMPA) and various ART regimens, thus validating the accuracy of routine program data from the EMR.

### Contraceptive use and ART

Approximately half of the women used contraceptives, one quarter of the women were on an EFV-based regimen, and there were nearly 13,000 incident pregnancies. The key finding was that women on EFV-containing regimens who were using implants to prevent pregnancy had increased weighted adjusted incident rate ratios (aIRRs) across all sampling methods: 1.9 (95% CI 1.6–2.4) in the EMR, 2.3 (95% CI 1.5–3.5) in chart review, and 3.2 (95% CI 1.8–5.7) in phone interviews. There was little to no evidence to support an increased pregnancy aIRR between DMPA and any of the ART regimens or between implants and non-EFV containing regimens.

### Patel and colleagues’ recommendations

Patel concluded the paper with three key recommendations: (1) countries can be more careful not to overreact to early reports of negative findings around drug-drug or drug-device interactions, (2) ensure individual women have information they need to make wise decisions, and (3) WLHIV are included in policy decision-making.

## Conclusions

### Data validation

This paper strengthens the literature by providing a resource for countries who may be seeking to understand the strengths and weaknesses of their own EMRs to guide decision-making in the face of changing global guidelines. This is a positive reflection on the significant donor investments and political will supporting development of health information systems in resource-constrained settings and highlights the strengths of programming at a centralized national level.

### Contraceptive use and ART

While the concurrent use of EFV-based ART and implants may not be relevant to the greatest proportion of WLHIV in their reproductive years, it remains a relevant issue to a large absolute number of women globally. In considering national program guidelines, it is important to consider equally the Family Planning (FP) and ART needs of women and ensure access to options, particularly in FP.

The authors focus primarily on the individual patient level needs in the face of national decision-making, but what they do not address in their conclusions is the very real challenge associated with national and sub-national supply chain management in resource-constrained settings. Standardized health systems and utilization of essential medicine lists mean not only that contraceptives and antiretrovirals are ordered by the millions but that there is a significant lead time between procurement and distribution of commodities in-country, usually in excess of 6 months, even when supply chain channels are well-established [7]. As a result, any decision to change ART regimens or relative availability and choice of contraceptives will take many months to be realized at the patient level. All stages of changing guidelines and supply chain management are further complicated by the reality that even when services are integrated at facility level, management of family planning and HIV programming at national and sub-national levels are infrequently managed in the same departments of the Ministry of Health. Any changes in one program that affect the other may require coordination across technical working groups and agreement across many more stakeholders, including donors.

These national level programming and supply chain challenges support Patel and colleagues’ recommendations that more caution could be taken before making changes to national guidelines. The ability of the EMR in Kenya to provide accurate estimates on the negative interactions between Efavirenz and hormonal implants provides clear direction for both identifying and reporting safety signals in the coming years.

## Data Availability

Not applicable
